# Conversion between 2 military combat-related injury coding systems

**DOI:** 10.1097/MD.0000000000010096

**Published:** 2018-03-09

**Authors:** Pengwei Hu, Fuxing Chen, Wang Chang, Tai Xie, Jinhui Liu, Zhiping An, Guoliang Chen, Xiaorong Liu

**Affiliations:** a Department of Health Service, Second Military Medical University, Shanghai; b Qingdao Convalescent Hospital, Qingdao; c Department of Navy Health Service, Second Military Medical University, Shanghai, China.

**Keywords:** code, combat, injury, MCIS, PC

## Abstract

Deployable medical systems(DEPMEDS) patient conditions (PCs) and Military Combat Injury Scale (MCIS) are 2 important military medical coding systems. However, both of them have defects when applied in military medical planning. Although each PC code contains information about treatment, intensity of care, treatment time, length of stay, and probability of disposition that is relevant to simulation, its description is too comprehensive and ambiguous to code historical military medical records. Therefore, conversion between PC and other medical coding systems applied in standard medical data is required when validity is required following simulation. The information linked to each PC code is based on subject matter expert opinion instead of standard medical data from the theater that need to be continuously updated depending on the results of medical data analysis. MCIS, a combat-related injury coding system, shows significant promise in coding real medical data, but it does not seek detailed information important for prediction and simulation unlike PCs. Therefore, MCIS cannot be used in planning tools directly. Thus, the effort to map MCIS to PCs is significant for medical logistic planning. We aim to identify whether conversion between PCs and MCIS is possible and to evaluate inter-coder reliability.

Three senior coders assigned all possible MCIS codes to 187 combat-related PC codes. The data records were structured based on an earlier study. Inter-rater reliability was measured by using Cohen's *k* statistic and percent agreement.

Low inter-rater reliability indicated the difficulty in conversion between PCs and MCIS.

The injury descriptors of PCs should be expanded by referring to new standard medical data. The existing MCIS codes need to be modified to include more information on treatment brief, treatment time, length of stay, and other key information, and historical data statistics need to be developed.

## 1 Introduction

Medical supplies demand and consumption estimation, medical resource allocation, and medical capacity deployment decisions are of great significance to military medical planning. The casualties’ medical condition is associated with different types of information, such as the consumption of medical materials, the use of healthy personnel, and the deployment of health capacity. The starting point of the predictions and decisions is the casualty's medical condition. Scientific summarizing and classification of possible injuries on the battlefield are the preconditions for prediction and decision-making. Currently, there are 2 widely used combat-related medical coding systems: deployable medical systems (DEPMEDS) patient conditions (PCs) and Military Combat Injury encoding and Scale (MCIS).^[1]^

In 1970s, to estimate medical supply, an expert panel including tri-services Department of Defense medical planners, operation research analysts, and physicians developed the U.S. army PCs system. In the early 1980s, the U.S. military subject matter experts enlarged the Army PCs and developed the DEPMEDS PCs, which define a group of patients with similar medical condition and treatment requirements, and can therefore be used in wartime medical resource allocation decisions. Frequencies of PCs were determined for U.S. Marines serving in Vietnam, consisting of 389 patient medical descriptions. The PCs were arranged in order from head to lower limbs, and each of them was marked with a 3-digit number ranging from 1 to 440. There were some gaps in numbers for future use. Among them, there are 313 PCs for conventional war encoding range 1 to 350, including 96 diseases, 146 noncombat injuries, and 187 wounded in action. There are 76 PCs for unconventional warfare, ranging from 351 to 440.^[2,3]^ Each PC was linked to a treatment brief, including the following information: the patient's description, complaints, brief treatment measures, and probabilities of return to duty, death, or evacuation. For inpatient injuries, the treatment brief also includes time of surgery, critical care, brief medical advice, brief physician orders in intermediate care units, and brief medical instructions in general care units. Owing to such detailed information, the PCs were widely applied in estimation, forecasting, and simulation programs such as Estimation Supply Program (ESP), Patient Workload Generator (PATGEN), and Tactical Medical Logistic+ (TML+). Although PCs are excellent for simulation, the PCs offer an overly broad and comprehensive description of the encoding that cannot be directly applied to historical data. This results in the need for conversion between PCs and other medical coding systems when validation using historical medical data in the theater becomes necessary to improve the estimation of medical resources and allocation. The descriptions of historical medical data are coded using International Classification of Disease (ICD) and not PCs. An U.S. army project linked the ICD-9 to PCs and developed conversion rules in 2001.^[4]^ The inter-rater reliability of this conversion was analyzed by Wojcik et al.^[2]^ The study found moderate support for the possibility of reliable conversion between PCs and ICD-9-CM diagnoses.

In November 2008, a U.S. Military Injury Score Summit developed an anatomy-based new combat-related injury score system. MCIS is based on thousands of historical medical combat injury descriptions, and it has redefined the combat-related body area and defined the classification standard of severity. The current version of MCIS includes a total of 269 codes.^[1]^ MCIS is simple and easy-to-use with 5 digits. It is effective for combat-related injury; the coverage rate was found to be 83% when tested in the sample data. MCIS has good inter-rater reliability, and the result is 91% to 93.5%. MCIS can also be associated with combat disability rating and linked to military functional incapacity of the casualties. Therefore, we believe that MCIS is a better tool to code combat-related medical data and can be widely used in the future. However, at present, MCIS is not directly related to the treatment measures, and each MCIS code does not have the same detailed information as PCs. Therefore, it cannot be directly applied to prediction and simulation modeling.

Thus, the effort to map MCIS to PCs or vice versa is significant for medical planning and establishing treatment guidance for each MCIS code. This study attempts to identify whether conversion between the 2 coding systems is possible and uses statistical methods to test inter-rater reliability.

## 2 Methods

### 2.1 Data sources

A total of 187 wounded-in-action codes extracted from 389 PCs codes and the MCIS coding guide manual were, respectively, provided to the 3 professional medical information coders.

All the 3 coders had experience in coding combat-related data and had participated in a project on establishing a combat-related injury coding system for the Chinese military. They were provided 2 hours of content training and taught the coding method for the 2 coding systems. Each coder was asked to assign all appropriate MCIS codes to each PC independently. Data records indicate how each coder mapped the MCIS to the PCs. The study focused on the conversion between 2 medical coding systems and did not involve patient records. So, the ethical protocol is not necessary.

### 2.2 Data analysis

We calculated both the percent agreement and Cohen's *k* statistic to measure inter-rater agreement using the method provided in Wojcik et al for mapping ICD-9 to PCs. MCIS includes 269 combat-related injury codes, providing the coders with 269 categories to choose from for each observation (PCs). To create a frequency table, mapping records were rearranged as follows: set arbitrary symbol “x.” The record consisted of the PC code plus coder 1, coder 2, and coder 3 variables, which were set equal to the specific MCIS code if the coder had assigned it and set equal to an arbitrary symbol, “x,” if the coder had not assigned it. This process was used to ensure that nonagreements were not treated by the software as missing data and omitted; (2) set pseudo-record. To ensure that all frequency tables were square (SAS requirement), which would result only if both raters used all of the same categories, we used the solution used in Wojcik et al, originally described by Uebersax. Each real data record was assigned a weight of 1, and each pseudo-record was assigned a very small weight of 1E-10. Table 1 provides an example of a record sheet.^[2,5,6]^

**Table 1:**
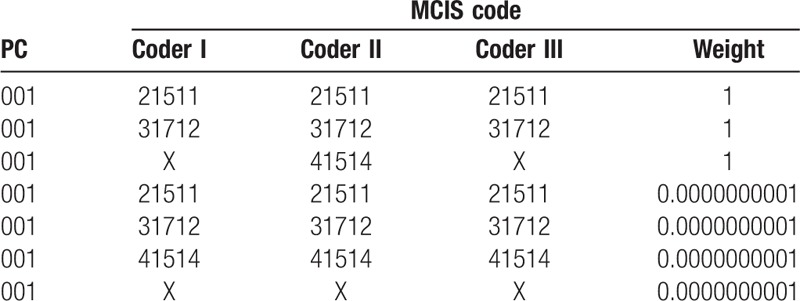
Example of an individual PC code mapping record.

Percent agreement is the ratio of the number of times the 2 coders agreed divided by the total number of mappings performed. The kappa statistic estimates the proportion of agreement among coders after the removal of random factors.

The percent agreement ranges from 0% to 100%, and the values for Cohen's *k* statistic range from −1 to 1, with 0 indicating complete agreement caused by random factors and negative values implying no agreement between raters. Generally, a Cohen's *k* statistic value between 0.81 and 1 implies almost complete agreement between raters, 0.61 to 0.8 implies high agreement, 0.41 to 0.6 implies moderate agreement, 0.21 and 0.4 implies the general degree of agreement, and <0.2 implies low agreement.

After discussion, the researchers decided to analyze inter-rater reliability agreement from the 3 viewpoints mentioned in Wojcik et al.^[2]^ The first viewpoint involved evaluating the inter-rater reliability of PCs individually. The second involved evaluating inter-rater reliability for identical mappings of individual PC codes. The third involved evaluating the agreement among all the records by coder pairs, without using individual mapping records.

## 3 Results

### 3.1 Inter-rater reliability of individual PC codes

A total of 183 MCIS codes were used to map the PC codes. Mean, standard deviation, mode, and quartile of *k* statistic, mean of MCIS codes matched, mean of MCIS codes used, and mean of percentage agreement for each PC code mapping record were recorded. The results are provided in Table 2. Overall, an average of 4.31 MCIS codes were used to map 1.35 PCs codes, with an average of 0.47% agreement. *k* Statistic for pairs of coders in mapping individual PCs codes ranged from −0.54 to 1. Summarized by coder pairs, the mean *k* statistic was 0.33, whereas the mean percent agreement was 41%. The mean *k* statistic of the 3-coder pairs groups was examined by the Mann–Whitney rank-sum test and was found to be statistically significant (*P* = .005 < .05).

**Table 2:**
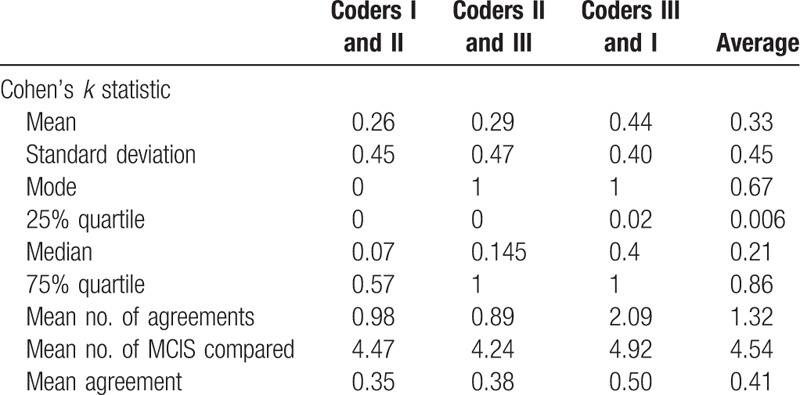
Inter-rater reliability of 3 coders summarized by coder pair.

### 3.2 Proportion of perfect agreements in all records

We defined the inter-rater agreement as the proportion of perfect agreements in all records. Coders I and II mapped an identical set of MCIS to 1 PC code for 42 records, accounting for 22.5% of the total number of records. Coders II and III mapped an identical set of MCIS to 1 PC code for 33 records, accounting for 22.5% of the total number of records, which was the same as coders I and III, as shown in Table 3. Overall, 33 (17.6%) mapping records have *k* statistic >0.6 in 3 coder pairs, 91 (48.7%) mapping records have *k* statistic between 0.4 and 0.6, and 63 (33.7%) mapping records have *k* statistic <0.4. For 9 PC codes, none of the 3 coders could map a set of MCIS codes, as shown in Table 4.

**Table 3:**
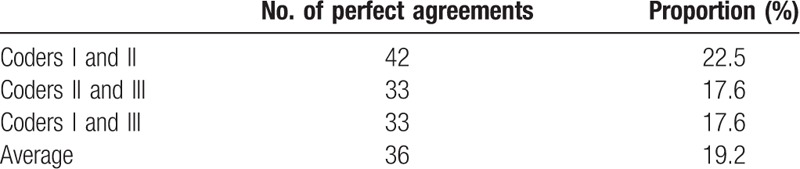
Proportion of perfect agreements in all records by coder pairs.

**Table 4:**
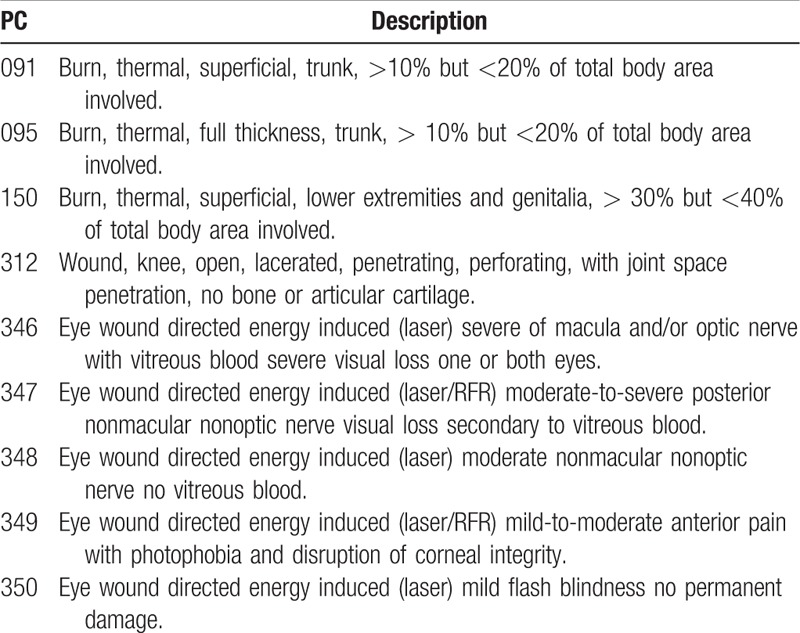
PC codes for which none of the 3 coders could map a set of MCIS codes.

### 3.3 Agreement without considering individual PC codes

For all the MCIS codes that the 2 coders selected as categories, the frequency of each PC code mapped was calculated. The calculation of inter-rater agreement was based on this rearrangement frequency table. Number of agreements, number of MCIS codes selected, percent agreement, and *k* statistic by coder pairs were calculated, as shown in Table 5. The mean percentage agreement was 62.6%, whereas the *k* statistic was 0.38.

**Table 5:**
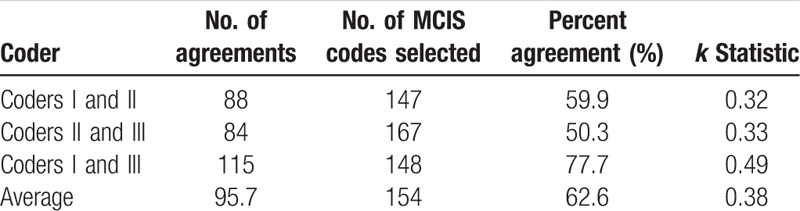
Inter-rater reliability without considering individual PC code summarized by coder pairs.

## 4 Discussion

Our analysis indicates low inter-rater reliability among the mapping records of coders, offering low support for conversion between the 2 medical coding systems. For viewpoint 1, the average percentage agreement of individual mapping records was 0.47, which is relatively the lower agreement level. According to the suggestion by Landis and Koch, *k* statistic between 0.4 and 0.75 represents good agreement beyond chance. The average *k* statistic of 3 coder pairs groups was 0.33, implying apparent low agreement. Although the mean *k* statistic of the 3-coder pairs groups was examined by the Mann–Whitney rank-sum test and was found to be statistically significant, the mean *k* statistic of the 2 groups was <0.4. For viewpoint 2, the average proportion of perfect agreements in all records summarized by coder pairs was 19.2%, which cannot support high agreement among coders. In addition, 9 PC codes were not mapped to any set of MCIS codes by the 3 coders. According to the description of these 9 PC codes and MCIS, the difference of burn severity classification between the 2 coding systems and lack of injury description by new weapons are the reasons for this. For viewpoint 3, the mean percentage agreement without considering individual PC codes was 62.6%, but the *k* statistic was 0.38, which is <0.4 and fails to support a good inter-rater reliability.

This result could have been obtained because the 2 medical coding systems were designed with different objectives. The original design objective of PC was to create groups of similar PCs requiring similar treatment, to estimate and predict medical supplies for battlefield. Therefore, the medical descriptions of PCs are very broad and comprehensive. MCIS is a combat-related injury coding and scale system centered on the anatomy that aims to code combat-related injury descriptions and score combat injury severity accurately. We analyzed the records for which the 3 coders mapped an identical set of MCIS to 1 PC code consistently (Table 6). Seven sets of MCIS codes were mapped to different PC codes implying that some of the MCIS code descriptions are broad and comprehensive. For the groups of records with average *k* statistic <0.4 that were analyzed, the 2 medical coding systems have the following differences in the classification of combat injuries. First, some of the combat injury severity classifications are different, reflected in injuries with loss of consciousness, maxillofacial fracture, and skin and soft tissue injuries. For example, for injuries with loss of consciousness, PCs divided the severity according to the time of consciousness loss, with intervals ranging from <2 hours, 2 to 12 hours, 12 to 24 hours, and >24 hours. Injuries associated with loss of consciousness include concussion, contusion, and head fractures (PC 001, PC 002, PC 003, PC 004, PC 005, and PC 006). Injuries involving loss of consciousness were classified in terms of head and neck body region in MCIS. Injury severity was described in terms of time interval of loss of consciousness: without loss or mild consciousness, 5 minutes to 1 hours, 1 to 6 hours, and >6 hours. Injuries associated with loss of consciousness include concussion, burns, axonal injuries, and other unexplained injuries (codes: 11710, 21711, 31712, 41713, and 41714).

**Table 6:**
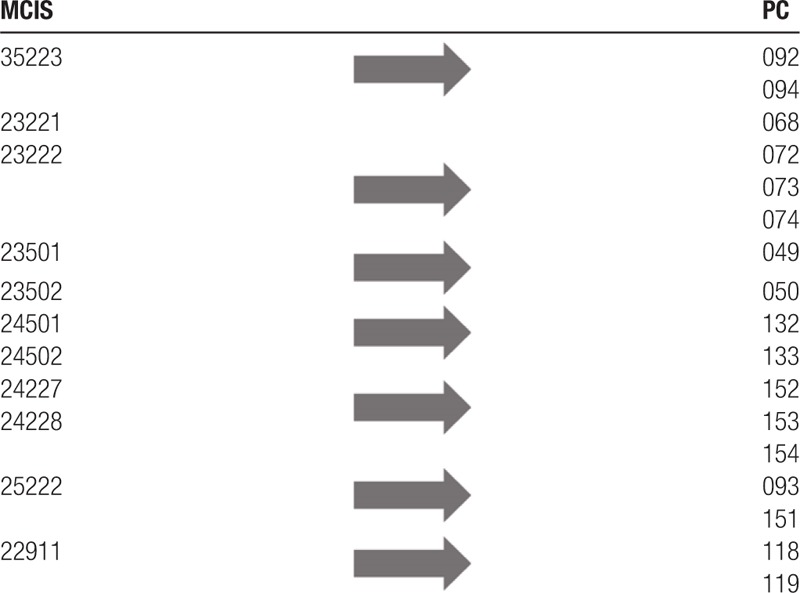
A set of MCIS codes mapped to various PC codes.

Moreover, injury description is significantly different between the 2 medical coding systems; the trauma-related PC codes resemble medical records with descriptions including injury details and treatment requirements, whereas MCIS is based on anatomy. For example, for vertebral fracture injury, patient condition of PCs is only described as vertebral fractures with or without spinal cord injured, whereas MCIS has a specific classification of vertebral fracture position, including thoracic fracture (32548), lumbar fracture (32551), and so on. For burns, PCs offer a detailed classification and description of the various parts of the body from shallow first-degree burns to third-degree burns, whereas MCIS does not describe the superficial first-degree burns. For chest wound, PCs offer a comprehensive description of injury, such as PC083, injury lung (blast crush) with pneumohemothorax severe-one lung with pulmonary contusion, and acute severe respiratory distress. MCIS classifies the chest organ in detail, and severity was classified according to air intake.

To simplify analyses, ICD-9-CM diagnosis codes were converted to 3-character codes, limiting coding outcomes to a maximum of 1000 categories in the prior study by Wojcik et al.^[2]^ ICD-10 diagnosis codes consist of letters and numbers, which can result in a maximum of 2600 categories of coding outcomes, if ICD-10 and PCs are converted. Introducing ICD-11 can result in a maximum of 268,280 categories. Moreover, changes in the classification rules influence the conversion. The most important ICD-9 diagnosis codes are selected as the main codes for multiple injury cases. A comprehensive ICD-10 diagnosis code is selected as the main code in the ICD-10 system. For example, skull fracture with intracranial hemorrhage is 1 code in ICD-9 (800.1), but it is coded separately as a main code-skull fracture and an additional code-intracranial hemorrhage in the ICD-10 system. Compared with ICD-9, ICD-10 classified injury and poisoning into the main category, defining neurological disorders, diseases of the eye and appendages, and mastoid diseases as separate categories. ICD-11 adds some more specific types of lesions and body region in the codes of injuries, introducing important classification features associated with the clinical presentation, treatment, and prognosis of the fracture (e.g., involvement of joints, organ, or vascular injury).^[7–11]^ If ICD-10 or ICD-11 is converted to PCs or vice versa, changes in the total number of categories and classification rules will affect the inter-rater reliability, but determining the specific impacts needs further study.

## 5 Conclusions

Each PC designates a group of patients with similar medical conditions to similar treatment requirements. Therefore, PC codes are too broad and ambiguous to code the diagnosis of patient records. Because it is not an anatomy-based coding system, it lacks a clear classification axis and attributes. MCIS is a completely anatomy-based coding system, whose classification axis, including body area, type of tissue, and injury severity, has proven to be specific for combat-related injury diagnosis. Before the operation, it is important that the medical commander plans the preparation and deployment of medical supplies, which requires the construction of a predictive model and the use of historical data for validation to improve accuracy. Planning factors are needed to build a predictive model, including treatment measures, duration of treatment, duration of hospitalization, probability of despositon, and so on. PCs consist of 389 codes, each of which is linked to treatment measures; operation room time; length of stay; and probability of return to duty, death, or evacuation. The current US military health simulation and decision-making software are based on PCs. To improve the prediction and decision-making treatment, validation using historical data is necessary. Historical data include the casualty rescue data in theater, the in-hospital patient record, and health resource allocation data. The diagnosis codes of historical data require a combat-related injury-specific coding system such as MCIS, owing to which the conversion between MCIS and PCs is very important. Inter-rater reliability was measured to access the level of agreement and feasibility of the mapping process. The results of our paper provided low support for the possibility of reliable conversion between 2 military medical coding systems. The results indicate that the conversion between MCIS and PCs will lead to deviation of the data, affecting the result of the planning model verification. The main reasons for this result are the different purpose and classification axis of the 2 coding systems.

Based on the analysis of the results, the paper makes the following recommendations. Future modifications to PCs need to be based on historical medical data, and adding or modifying entries on the basis of detailed assessment of injury type or severity. The number of items of PC codes needs to be increased because only 183 codes of MCIS were selected to map PC codes, and 87 were not used, suggesting that PCs may not cover some combat-related injuries. Although the MCIS has good pertinence and adaptability when applied to combat injury data and can cover most wounds in action, it also has defects, such as lack of subdivision of eye wounds, description of metal foreign body retention, subdivision of joint injury, and injury caused by new weapon.

MCIS codes are currently not linked to treatment measures, which may prevent MCIS from being directly applied to simulation or decision-making software. We recommend adding additional descriptions, such as whether crush injuries can be repaired and whether surface injuries require large debridement. In addition, MCIS also has some comprehensive descriptions, which should preferably be subdivided. Owing to the lack of one-to-one mapping between the 2 medical coding systems, establishing diagnostic-related groupings may be effective. With this procedure, the primary diagnosis can identify a set of PCs, following which other patient information, such as secondary diagnosis or complications, can be determined by a decision tree for a specific PC.

If we treat the PC codes as the patient's medical data, this analysis can be regarded as an analysis of inter-rater reliability of MCIS codes in a test data. The low inter-rater reliability implies that MCIS may need modification. We think that the best solution is the modification of MCIS, such as referring to PC treatment abstract approach, adding treatment briefs to each MCIS code, or designating diagnostic-related groups based on MCIS. In addition, PCs deal with nonbattle injury, disease, and wounded in action, and are not part of a medical coding system for combat-related injury only. MCIS is a combat-related coding system for injury, which cannot be used to code disease, and there is no evidence to indicate that it can be applied to nonbattle injury. It is necessary to broaden the codes to describe nonbattle injury and disease in theater in MCIS, which would make it possible to apply MCIS in medical simulation or decision-making programs.^[12]^

## 6 Limitations

The accuracy of mapping results has not been evaluated in this paper. The subjectivity of the coder itself may have led to inaccurate mapping results and affected the inter-rater reliability. As the official guidebook of both PCs and MCIS were not available, we compiled the guidebook according to code list and references ourselves before the mapping, to ensure that conversion did not confirm to the original intentions of the developers of both PCs and MCIS.
